# Introducing clinical pharmacy specialists into interprofessional primary care teams: Assessing pharmacists’ team integration and access to care for rural patients

**DOI:** 10.1097/MD.0000000000026689

**Published:** 2021-09-24

**Authors:** Megan B. McCullough, Anna Zogas, Chris Gillespie, Felicia Kleinberg, Joel I. Reisman, Ndindam Ndiwane, Michael H. Tran, Heather L. Ourth, Anthony P. Morreale, Donald R. Miller

**Affiliations:** a Center for Healthcare Organization and Implementation Research, VA Bedford Healthcare System, Bedford, MA; b University of Massachusetts, Lowell, Zuckerberg School of Health Sciences, Department of Public Health, Lowell, MA; c Center for Healthcare Organization and Implementation Research, VA Boston Healthcare System, Boston, MA; d Pharmacy Benefits Management Services, Veterans Health Administration Central Office, Department of Veterans Affairs, Washington, DC; e University of Massachusetts, Lowell, Center for Population Health, Department of Biomedical and Nutritional Sciences, Lowell, MA.

**Keywords:** access to care, clinical pharmacists, comprehensive medication management, interprofessional care teams, primary care

## Abstract

Clinical pharmacy specialists (CPS) were deployed nationally to improve care access and relieve provider burden in primary care.

The aim of this study was to assess CPS integration in primary care and the Clinical Pharmacy Specialist Rural Veteran Access (CRVA) initiative's effectiveness in improving access.

Concurrent embedded mixed-methods evaluation of participating CRVA CPS and their clinical team members (primary care providers, others).

Health care providers on primary care teams in Veterans Health Administration (VHA).

Perceived CPS integration in comprehensive medication management assessed using the MUPM and semi-structured interviews, and access measured with patient encounter data.

There were 496,323 medical encounters with CPS in primary care over a 3-year period. One hundred twenty-four CPS and 1177 other clinical team members responded to a self-administered web-based questionnaire, with semi-structured interviews completed by 22 CPS and clinicians. Survey results indicated that all clinical provider groups rank CPS as making major contributions to CMM. CPS ranked themselves as contributing more to CMM than did their physician team members. CPS reported higher job satisfaction, less burn out, and better role fit; but CPS gave lower scores for communication and decision making as clinic organizational attributes. Themes in provider interviews focused on value of CPS in teams, relieving provider burden, facilitators to integration, and team communication issues.

This evaluation indicates good integration of CPS on primary care teams as perceived by other team members despite some communication and role clarification challenges. CPS may play an important role in improving access to primary care.

## Abstract 2

Supplemental Digital Content is available in the text

## 1 Introduction

Improving access to primary care for rural Americans is a critical issue for the US healthcare system and a priority area for the Veterans Health Administration (VHA).^[1]^ Access to care is hindered by a national shortage of primary care doctors, increased rates of primary care clinician burnout,^[2,3]^ and patients’ need for labor-intensive comprehensive medication management (CMM) and disease state management.^[4–7]^ CMM is individualized care that addresses all aspects of patients’ medication use (prescription, nonprescription, alternative, traditional, vitamins, and nutritional supplements) for safety and effectiveness.^[8]^ CMM decisions are increasingly complex, and medications are involved in 80% of patient care encounters.^[9]^ There is high prevalence of complex chronic medical conditions in Veterans and they often have more severe chronic disease than non-veterans, which further increases demand on primary care physicians.^[10–12]^ Including a clinical pharmacy specialist (CPS) in primary care teams can increase patient access and relieve provider burden.^[13–15]^

To address critical system-wide issues of access to care and CMM for rural-dwelling Veterans, the VHA's Office of Rural Health and Pharmacy Benefits Management Services Clinical Pharmacy Practice Office implemented a nationwide initiative to improve rural Veterans’ access to care and ease primary care provider burden by adding CPS to primary care. The CPS is an advanced practitioner with prescriptive privileges and the ability to collaboratively and independently engage in CMM.^[14,15]^ CPS are serving in interprofessional primary care teams, which are referred to as Patient Aligned Care Teams (PACT) in the VHA. On these teams, CPS contribute substantially to CMM, provide significant clinical services that complement the work of other PACT providers (OPP), and improve the overall capacity of the primary care clinic,^[16]^ in providing same day access, laboratory assessments, and management of adverse drug reactions.^[17]^ They contribute to improvements in intermediate clinical outcomes and overall population health.^[18–24]^

The Clinical Pharmacy Specialist Rural Veteran Access (CRVA) initiative included a mixed methods program evaluation. As a field, clinical pharmacy is expanding, and CPS are filling needed and necessary gaps in care as well as relieving provider and physician burden. However, few studies have examined CPS integration on primary care teams from the perspectives of both the CPS and other members of their interprofessional teams. This mixed methods project identifies key lessons learned from the integration of CPS into primary care from the clinician, clinical team member, and pharmacists’ perspectives.

## 2 Methods

The evaluation design is a concurrent embedded design,^[25]^ in which both qualitative and quantitative arms of the evaluation run concurrently. Informed by the Reach, Effectiveness, Adoption, Implementation, Maintenance (RE-AIM) framework,^[26]^ the evaluation assesses the initiative's reach (the extent to which it reached patients), its adoption by staff (integration of the CPS into existing interprofessional care teams), and the fidelity of the staff's adoption of the program to how it is intended to be implemented (e.g., the extent to which CPS are practicing to the top of their scope). The project concurrently combines encounter data, survey results, and qualitative interviews. The evaluation began at the start of the CRVA initiative in October 2017; we report here on results based on data collected through March 2020. This evaluation was reviewed by the Institutional Review Board at the VA Bedford Healthcare System in Bedford, MA, and the IRB determined that this evaluation to fit in the category of quality improvement (QI). In VHA, QI studies are not subject to IRB oversight. However, elements of informed consent were adhered to and confidentiality of participants was observed.

### 2.1 Encounter data methodology

Visits with CPS were identified from the VHA Corporate Data Warehouse (CDW), a repository of national VHA data that contains comprehensive information related to medical encounters as well as clinical, financial, and demographic patient data. *Clinical visits* were those occurring face-to-face, group, home based, and telemedicine. *Other medical encounters* included the performance of related functions such as consultations.

### 2.2 Survey evaluation

The survey evaluation component is a fixed panel approach. CPS who were hired for the CRVA initiative and served on the program for at least 6 months were identified. We contacted the program's local site champions (e.g., pharmacy leadership) who provided lists of the health professionals who were members of clinical teams with the CPS. All eligible CPS and members of their primary care teams, whom we refer to as OPP, were invited to participate in the survey. To improve participation, potential participants received consistent e-mail reminders with a survey link and at least one instant message reminder.^[28]^ We report here on findings derived from the cross-sectional baseline surveys.

Data were collected via self-administered questionnaires on the web-based REDCap system. The survey's principal measure of integration is a modified version of the Medication Use Process Matrix (MUPM) developed by Farrell et al.^[29]^ This assesses the perceived role of providers in CMM across 5 key domains (evaluation & management, medication monitoring, medication review, documenting care, and medication education).^[30]^ Item scores range from 0 (no contribution) to 3 (major contribution), so higher scores indicate greater integration in primary care teams. The MUPM was administered to CPS and to OPP, assessing their own roles in CMM as well their perceptions of the CPS contributions. Standardized composite scale scores, computed as unweighted averages across the five domains, were computed and used in analysis of variation in MUPM across VHA stations.

Additional survey measures include a CPS-Provider collaboration scale adapted from the Frequency of Interprofessional Collaboration Instrument developed by Van et al.^[31]^ This evaluates CPS-OPP collaboration and the frequency of interactions that comprise actual collaborative behavior. The survey also includes a measure of Organizational Attributes of Practice Settings developed by Ohman-Strickland et al.^[32]^ It assesses organizational attributes (e.g., leadership and practice culture) and internal resources available to practice teams as care providers in 4 domains: (1) communication, (2) decision making, (3) stress/chaos, and (4) history of change. The survey also assesses: perceptions of job responsibilities relative to professional expectations, that is, performing tasks that are below, above, or well-matched to one's training and competence; job satisfaction; professional burn-out.^[33]^

Response differences between CPS and other providers were evaluated by comparing CPS with each other provider group in turn. We used the Mann--Whitney *U* test for ordinal scales and the Chi-square test for dichotomous and multi-category variables.

### 2.3 Qualitative interviews

The qualitative team included 2 PhD anthropologists (MM, AZ) and 1 PhD sociologist (CG). CPS and OPP who completed the survey served as a candidate pool for interviews. We used a purposive sampling approach. First, we used survey data and data from the CRVA project to identify CPS (see Supplemental materials 1, http://links.lww.com/MD/G289 for interview recruitment). We then identified OPP who worked with these CPS who had also completed the survey and invited them to be interview. This resulted in 6 CPS interviews and 16 OPP. A total of 22 interviews were conducted.^[27,34]^

Interview guides were informed by the RE-AIM framework, which was specifically designed to evaluate program elements to improve effective evidence-based interventions.^[35,36]^ In addition, relevant literature on CPS roles and interprofessional teams in primary care was used in generating the interview guide. CPS were asked to discuss how they set up their practice, access to care for patients, contributions to CMM (including disease state management and population health), and team integration. OPP were asked to share their experience working with CPS and their perception of CPS contributions to teamwork, workload, workflow, and patient care.

Interviews were conducted by telephone and ranged 15 to 60 minutes with an average of 30 minutes. Interviews were audio recorded and transcribed verbatim. We used a process of critical review and consensus-building to develop a codebook that included deductive codes reflecting key concepts from the surveys (e.g., the MUPM's five domains of medication use processes), RE-AIM, and the scientific literature as well as inductive codes.^[25]^ Deductive codes focused on team dynamics, team roles, and pharmacist role in CMM. Inductive codes captured concepts related to interprofessional communication, points of possible conflict and comfort with expansion of clinical pharmacy services^[25]^ (see Supplemental materials 2, http://links.lww.com/MD/G290 for Codebook). NVivo was used for qualitative data analysis and to facilitate comparisons between team members’ coding. Discrepancies in coding were resolved with final judgement by the qualitative lead and project PI (MM). On the basis of our concurrent embedded design, the mixed methods data were triangulated through ongoing synthesis of data which informed each the qualitative and quantitative arms of the study.^[25]^

## 3 Results

A total of 496,323 medical encounters were performed by CPS in the CRVA program, from the time of its inception in October 2017 through March 2020. Encounters increased from 59,558 in the first year to 187,383 in the most recent year of the initiative. Of all encounters, 86.5% were clinical visits with patients; 71.8% were performed in rural areas. For VHA, rural areas are defined, per the U.S. Census definition, as any population, housing or territory not in an urban area.^[37]^

One hundred twenty-four CPS and 1177 OPP responded to baseline surveys, with participation proportions of 98% and 70%, respectively. OPP team member respondents included 243 primary care medical doctors (PCP-MD), 156 primary care physician's assistants or nurse practitioners (PCP-PA/NP), and 241 registered nurse clinical coordinators (RNCC). There were 537 additional primary care team members (ATM), including 113 registered nurses, physician assistants, or nurse practitioners (N/PA/NP), 51 social workers (SW), and 239 licensed practical nurses (LPNs). Some demographic differences were observed among survey participants in comparisons of CPS with OPP (Table 1). CPS were considerably younger with less time in VA service and less time since their last professional degree. Most CPS were under the age of 40 years and had less than 6 years of postgraduate experience. CPS also were more often female and less often Veterans as compared to PCP physicians. From this sample of respondents, we conducted 22 interviews, 6 among primary care CPS, and 16 among OPP.

**Table 1:** Survey Respondents of PACT (Primary Care) Team Members.

	CPS	PCP MD	PCP PA/NP	RNCC	ATM
n	124	243	156	241	537
Age, yr					
20–29	43 (37.1%)	0 (0.0%)	0 (0.0%)	6 (2.8%)	19 (4.3%)
30–39	48 (41.4%)	16 (7.7%)	21 (15.6%)	31 (14.5%)	74 (16.6%)
40–49	18 (15.5%)	61 (29.3%)	28 (20.7%)	54 (25.2%)	130 (29.1%)
50–59	5 (4.3%)	74 (35.6%)	61 (45.2%)	79 (36.9%)	152 (34.0%)
60–69	2 (1.7%)	49 (23.6%)	25 (18.5%)	43 (20.1%)	66 (14.8%)
70 or older	0 (0.0%)	8 (3.8%)	0 (0.0%)	1 (0.5%)	6 (1.3%)
Sex					
Male	27 (23.3%)	113 (54.9%)	31 (22.6%)	27 (12.7%)	75 (16.9%)
Female	89 (76.7%)	93 (45.1%)	106 (77.4%)	186 (87.3%)	369 (83.1%)
Military Veteran					
no	106 (89.8%)	178 (85.2%)	113 (81.9%)	168 (78.9%)	350 (78.0%)
yes	12 (10.2%)	31 (14.8%)	25 (18.1%)	45 (21.1%)	99 (22.0%)
Years since last professional degree					
<6	61 (56.0%)	2 (1.1%)	25 (20.0%)	27 (15.4%)	50 (15.5%)
6–12	28 (25.7%)	20 (11.0%)	39 (31.2%)	61 (34.9%)	87 (26.9%)
13–20	15 (13.8%)	43 (23.6%)	34 (27.2%)	34 (19.4%)	66 (20.4%)
20 or more	5 (4.6%)	117 (64.3%)	27 (21.6%)	53 (30.3%)	120 (37.2%)
Years in VA Service					
<6 years	92 (74.2%)	144 (59.3%)	102 (65.4%)	134 (55.6%)	318 (59.2%)
6 or more	32 (25.8%)	99 (40.7%)	54 (34.6%)	107 (44.4%)	219 (40.8%)

ATM = Additional team members; CPS = Clinical pharmacy specialists; PACT = Patient Aligned Care Teams; PCP MD = Primary care providers - medical doctors; PCP PA/NP = Primary care providers - physician's assistants or nurse practitioners; RNCC = Registered nurse clinical coordinators.

**Figure 1:**
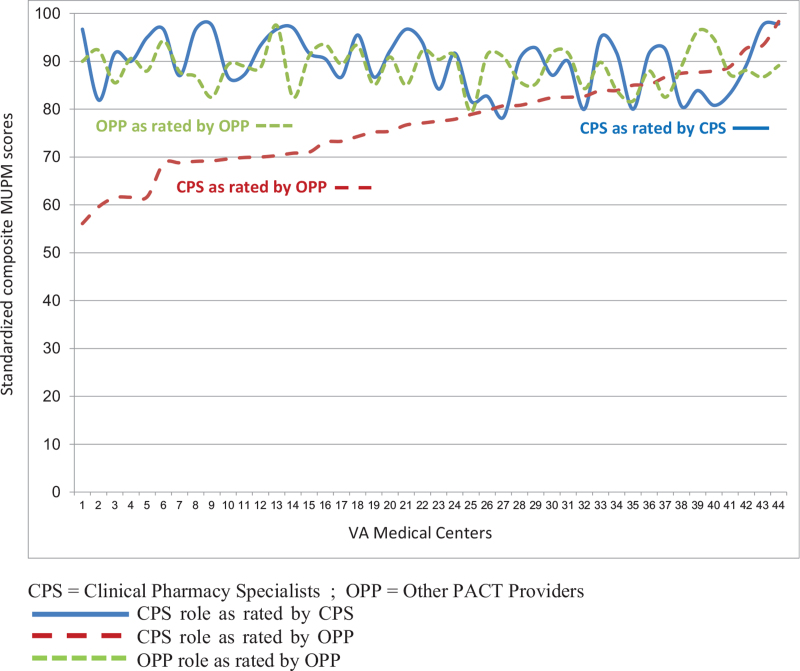
Variation in Medication Use Process Matrix scores across the National VHA. X-axis title: Scores from 44 VHA sites with at least 3 respondents ranked by CPS role as rated by OPP. Y axis title: Average standardized composite scale scores. CPS Role as Rated by CPS; CPS Role as Rated by OPP; OPP Role as Rated by OPP. CPS = Clinical Pharmacy Specialists; OPP = Other PACT Providers.

Findings from the MUPM for primary care providers are presented in Table 2. CPS and all groups of OPP agreed on the high level of contributions that CPS make in all 5 domains of CMM (Section A). Mean rating scores for the CPS role ranged from 2.3 to 2.9 (3.0 is the highest score). Scores were particularly high for medication review and documenting care. Providers from 3 of the OPP groups also rated themselves in terms of their own CMM contributions (Section B). PCP-MDs and PCP-PA/NPs considered themselves to make major contributions to all domains of CMM, except for medication education. In fact, their rating of their own contribution in evaluation and management was higher than the CPS rating of themselves, and PCP providers considered their own role on 4 of 5 of the domains to be greater than the role that they considered for the CPS. RNCCs consider their own contribution to be lower across all MUPM domains, while assigning high contributions to CPS. In examination of how scores varied across VA medical centers (Fig. 1), we found consistently high average scores for CPS contributions and only modest variation in how both CPS and other providers rate themselves. There was, however, notable variation among medical centers in how other providers score CPS on their contributions, with average composite scores ranging from 56 to 98 on a scale of 0 to 100.

**Table 2:** Integration of PACT clinical pharmacy specialists as measured by the medication use process matrix.

A. CPS role perception by	CPS	PCP MD	PCP PA/NP	RNCC
Evaluation and management	2.67 (0.33)	2.19 (0.69)^∗∗∗^	2.33 (0.64)^∗∗∗^	2.49 (0.54)^∗^
Medication monitoring	2.83 (0.35)	2.31 (0.74)^∗∗∗^	2.41 (0.70)^∗∗∗^	2.51 (0.61)^∗∗∗^
Medication review	2.86 (0.36)	2.54 (0.73)^∗∗∗^	2.63 (0.63)^∗∗^	2.80 (0.49)
Documenting care	2.87 (0.41)	2.31 (0.85)^∗∗∗^	2.47 (0.82)^∗∗∗^	2.59 (0.76)^∗∗^
Medication education	2.56 (0.44)	2.33 (0.66)^∗^	2.39 (0.61)^∗^	2.53 (0.55)
CPS role perception by:	CPS	N/NP/PA	SW	LPN
Evaluation and management	2.67 (0.33)	2.55 (0.52)	2.50 (0.68)	2.49 (0.62)
Medication monitoring	2.83 (0.35)	2.55 (0.63)^∗∗∗^	2.49 (0.79)^∗∗^	2.48 (0.70)^∗∗∗^
Medication review	2.86 (0.36)	2.80 (0.50)	2.79 (0.64)	2.65 (0.63)^∗∗^
Documenting care	2.87 (0.41)	2.54 (0.74)^∗∗∗^	2.58 (0.74)^∗∗^	2.54 (0.76)^∗∗∗^
Medication education	2.56 (0.44)	2.44 (0.60)	2.58 (0.60)	2.47 (0.62)
B. Own role perception by:	CPS	PCP MD	PCP PA/NP	RNCC
Evaluation and management	2.67 (0.33)	2.94 (0.16)^∗∗∗^	2.96 (0.12)^∗∗∗^	1.63 (0.59)^∗∗∗^
Medication monitoring	2.83 (0.35)	2.81 (0.29)	2.88 (0.24)	1.89 (0.66)^∗∗∗^
Medication review	2.86 (0.36)	2.79 (0.40)	2.89 (0.28)	1.87 (0.83)^∗∗∗^
Documenting care	2.87 (0.41)	2.87 (0.37)	2.97 (0.18)^∗^	2.40 (0.75)^∗∗∗^
Medication education	2.56 (0.44)	2.08 (0.49)^∗∗∗^	2.19 (0.44)^∗∗∗^	1.85 (0.54)^∗∗∗^

Scale scores range from 0 (no contribution) to 3 (major contribution). Mean scale scores (standard deviation). CPS = Clinical pharmacy specialists; LPN = Licensed practical nurses; N/NP/PA = Other nurses, physician assistants, or nurse practitioners; PACT = Patient Aligned Care Teams; PCP MD = Primary care providers - medical doctors; PCP PA/NP = Primary care providers - physician's assistants or nurse practitioners; RNCC = Registered nurse clinical coordinators; SW = Social workers. *P*-value from Mann--Whitney U tests in comparison with CPS: ^∗^ 0.05--0.01; ^∗∗^ 0.009--0.001; ^∗∗∗^ <.0001.

Findings from the interviews support the widely held perception that CPS are making substantial contributions to patient care (Table 4). Interprofessional team members feel that CPS have taken on key areas in CMM, such as patient education and counseling. CPS see themselves as being critical team members, working effectively with patients and improving CMM in primary care overall. CPS and OPP similarly see CPS as relieving primary care providers of certain medication management tasks and therefore adding value to primary care teams (see Table 4).

Other measures from the survey also suggest successful integration of primary care CPS in team care (Table 3). Inter-professional collaboration (information sharing and consultative interactions) was indicated by CPS as occurring 3 to 4 times in the past week on average. There appeared to be no differences in the frequency of interactions reported by the CPS and either the PCP MD or the PCP PA/NP, but RNCCs reported less frequent interactions with the CPS. With respect to organizational attributes of the primary care practice setting, CPS reported lower average scores for communication as compared to the three OPP groups, lower scores for decision making as compared to PCP MD and RNCC, and lower scores for stress/chaos than PCP MD. This indicates that, compared with other primary care providers, CPS perceive more problems with conflict resolution, tension among team members, lack of consensus building, and other leadership deficits. OPP stated in interviews (Table 4) that their burden of care is relieved as CPS take on CMM, especially with complex patients. CPS likewise see themselves as increasing access to care in providing critical support for their primary care teams.

**Table 3:** Other survey measures for PACT clinical pharmacy specialists and other PACT providers.

	CPS	PCP MD	PCP PA/NP	RNCC
n	124	243	156	241
Interprofessional Collaboration (1–5)^†^	3.27 (0.94)	3.05 (1.12)	3.06 (1.10)	2.77 (1.15)^∗∗∗^
Organizational Attributes of Primary Care (1–5)^†^				
Communication	3.73 (0.84)	4.13 (0.75)^∗∗∗^	4.01(0.81)^∗∗^	4.17 (0.77)^∗∗∗^
Decision Making	3.77 (0.90)	4.05 (0.94)^∗∗^	3.84 (1.08)	4.04 (0.99)^∗∗^
Improvements	3.52 (0.72)	3.60 (0.80)	3.35 (0.88)	3.63 (0.82)
Stress/Chaos	3.20 (0.79)	3.42 (0.90)^∗∗^	3.34 (0.83)	3.23 (0.88)
History of Change	3.34 (0.61)	3.44 (0.74)	3.23 (0.81)	3.46 (0.77)
Job Satisfaction (1--6)^†^	4.82 (0.71)	3.96 (1.01)^∗∗∗^	4.09 (0.83)^∗∗∗^	4.33 (0.94)^∗∗∗^
Burn Out (1–7)^†^	3.74 (1.47)	4.52 (1.92)^∗∗∗^	4.24 (1.83)^∗^	3.82 (1.81)
Burn out from work at least once per week	5.73 (0.78)	6.12 (0.75)^∗∗∗^	6.00 (0.73)^∗∗^	5.87 (0.77)
Time spent on tasks that are well matched to training				
<50%	8.3%	25.8%	9.1%	25.6%
50–75%	36.7%	35.5%	30.1%	31.1%
> 75%	55.0%	38.7%^∗∗^	60.8%	43.4%

PACT = Patient Aligned Care Teams; CPS = Clinical pharmacy specialists; PCP MD = Primary care providers - medical doctors; PCP PA/NP = Primary care providers - physician's assistants or nurse practitioners; RNCC = Registered nurse clinical coordinators. P value from Mann--Whitney U tests in comparison with CPS: ^∗^ 0.05--0.01; ^∗∗^ 0.009--0.001; ^∗∗∗^ <0.0001.

† Range of scale or item scores. Mean scale scores (standard deviation).

**Table 4:** Qualitative results.

Comprehensive Medication Management
*Clinical Team Members*
“I think the providers don’t have the time to go over all the little details. So when they’re [CPS] processing the meds, I think it's a good time when they do education with them [patients] and hopefully figure out what else might be needed…Like the Veteran may need a pill box and they may need something else, and they may not be able to manage their meds. The more patients [CPS] talk to and they can catch these problems.” [Primary Care Provider, team member]
*Clinical Pharmacists*
“And then as medications are being changed, they’re [patients] kind of taking more initiative and more ownership of their disease state. And so, they [patient] feel more confident in managing their disease states whether it's diabetes, blood pressure, whatever the case it…the patient is like, ‘I couldn’t have done this without my clinical pharmacy specialist.”’ [Clinical Pharmacy Specialist]

Value of CPS
*Clinical Team Members*
“The PharmD takes some of that [pressure] off the physician and can work with nurses to try to get a better plan of care for the patient. To try to educate them [patients] better for their disease processes—a lot of them [patients] lack education in a huge way and that's a sad thing.” [LPN, team member]
*Clinical Pharmacists*
“… They [providers] enjoy having someone to manage more of the complex patients, more time-consuming patients, high-risk patients, and then having that assistance when necessary…the clinics that I work with, the providers may be a little bit overworked…And so us coming in and providing any assistance is great. Also, us taking those high-risk patients or patients that require more specialized follow-up, they really enjoy that.” [Clinical Pharmacy Specialist]

Access to Care
*Clinical Team Members*
“And they’re [CPS] helping to be able to manage those more efficiently so that then I don’t have to. And it makes my job then much more efficient. When I can just…get them started on something, and then I don’t have to see them back in two weeks, or four weeks or whatever, and then see them back in another month.” [Primary Care Provider - Physician Assistants, team member]
*Clinical Pharmacists*
“It's been a struggle for the team…just the amount of walk-ins we have to deal with…the RN can utilize me [CPS] if it's an acute problem that the Veteran is having but it may be related to their chronic disease…She’ll ask me to come in instead of the provider, and we can look and see if we can make an adjustment. And so that frees up the provider to stay in her clinic seeing her scheduled patients.” [Clinical Pharmacy Specialist]

Job satisfaction was generally higher among CPS as compared to the 3 OPP groups (Table 3). It was particularly low for PCP MD and PCP PA/NP and these groups also reported significantly more job burn-out than did CPS. CPS also reported spending more of their working time on tasks that were well-matched to their training as compared to PCP MD and RNCC.

## 4 Discussion

CPS in the CRVA program make major contributions in all areas of CMM. This includes medication evaluation, management, monitoring, review, education, documentation, and population management (i.e., use of dashboards, etc, to track high risk patients and medications).^[14,16]^ Through CMM, CPS ensure each patient's medications (i.e., prescription, nonprescription, alternative) are assessed to determine that the medications are appropriate, effective, and safe.^[38]^ CPS are also expert in disease state management where they work to prevent or minimize the effects of chronic disease through integrated care for patients. High volume areas such as diabetes, hypertension, anemia, chronic obstructive pulmonary disease, heart failure, hyperlipidemia, pain, anticoagulation, and hepatitis C are key areas where CPS engage in disease state management in VHA and in other health care systems.^[16]^

Clinic team members reported in interviews that CPS were valuable and able to relieve provider burden in key areas of CMM, such as helping complex diabetes patients reach therapeutic goals. CPS also made sure they were available for informal and formal consults with their clinical team members. In agreement with survey findings, CPS integration was strong. Interview data indicated that integration was facilitated by the willingness of CPS to reach out to their primary care team members and educate them about CPS expertise and abilities. CPS also used population health tools (disease state dashboards) to find patients in need of follow-up as well as formal and informal networking for patient referrals from clinical team members. All these actions by CRVA CPS sought to build trust and acceptance and helped facilitate successful implementation of the initiative.

The CRVA program has scaled up rapidly in placing CPS in clinical teams, providing nearly half a million medical encounters over a 3-and-a-half-year period. Our evaluation of this primary care focused intervention found that CPS integration into established primary care teams has been successful, based on service utilization and providers’ perceptions. Such public health initiatives in VHA, and evaluations of their uptake in clinics, have increasing relevance as health systems adopt and increase team-based care as a way of extending and improving primary care. Furthermore, the increasing role of the CPS in comprehensive medication and disease state management is generalizable to other care settings (e.g., pain management; mental health) and other health care systems.

There are some challenges to CPS’ integration. There were lower scores on the communication part of the interprofessional team collaboration measurement. A small number of CPS noted in interviews that they were not invited initially to team meetings. Situations like this may have led to lower communication scores. We also observed differences among clinical team members in perceptions of CPS contributions in certain domains of CMM and this points to areas of practice where the roles are still being negotiated. For example, in the survey, primary care physicians perceived themselves as major contributors to evaluation and management, and they rank CPS lower than CPS rank themselves. However, in interviews, clinical team members note that CPS are experts at CMM, and this is greatly valued in terms of saving primary care provider effort and time. Minor differences are to be expected in a large implementation initiative and this variation may also reflect the growing pains of the process of integration itself.

This project has strengths and limitations. Nearly all of the CPS identified from the CRVA initiative and recruited actually participated in the survey and we successfully recruited 70% of other providers. These participation rates are very good as compared to other provider surveys and suggest that response bias was low.^[39,40]^ Similarly, recruitment for interviews was successful. Although the qualitative interviews were modest in size, we interviewed CPS and members of their clinical teams until no new knowledge emerged from the data.^[27,41]^ Analyzing pharmacist and interprofessional clinical team members views’ concurrently is a strength of this project, as it provides greater insight into the process of integration, which involves both groups. A limitation of this project is that it does not include formal cross-site comparisons. We also did not have observed measures of team integration to complement the reports of team members.

## 5 Conclusion

Clinical pharmacy is an area that is expanding in the primary care because CPS make such valuable contributions to increasing access to care by providing CMM, which also relieves provider burden.^[42]^ In the CRVA initiative, it is apparent that CPS are valued by their clinical team members and are being successfully integrated into clinical teams. For healthcare systems considering increasing their team by including CPS, this quality improvement project offers insight into how interprofessional integration happens, the value of the CPS as a provider who extends access to CMM in primary care teams.

## Acknowledgments

Authors would like to thank all evaluation participants for their generosity and time. We would like to thank colleagues at CHOIR for their feedback and offer a special thanks for the support of the VHA Office of Rural Health and the Clinical Pharmacy Practice Office.

## Author contributions

**Conceptualization:** Megan B. McCullough, Donald R. Miller.

**Data curation:** Megan B. McCullough, Donald R. Miller.

**Formal analysis:** Megan B. McCullough, Anna Zogas, Chris Gillespie, Joel I. Reisman, Ndindam Ndiwane, Donald R. Miller.

**Funding acquisition:** Megan B. McCullough.

**Investigation:** Megan B. McCullough, Anna Zogas, Chris Gillespie, Donald R. Miller.

**Methodology:** Megan B. McCullough, Chris Gillespie, Joel I. Reisman, Michael H. Tran, Donald R. Miller.

**Project administration:** Megan B. McCullough.

**Resources:** Megan B. McCullough, Michael H. Tran.

**Supervision:** Megan B. McCullough.

**Writing – original draft:** Megan B. McCullough.

**Writing – review & editing:** Anna Zogas, Chris Gillespie, Felicia Kleinberg, Joel I. Reisman, Ndindam Ndiwane, Michael H. Tran, Heather L. Ourth, Anthony P. Morreale, Donald R. Miller.
